# Exploring haemodynamics of haemodialysis using extrema points analysis model

**DOI:** 10.1186/1742-4682-10-33

**Published:** 2013-05-16

**Authors:** Mohamed Tarek Eldehni, Aghogho Odudu, Christopher William McIntyre

**Affiliations:** 1 Division of Vascular Medicine, Faculty of Medicine and Health Sciences, School of Graduate Entry Medicine and Health, The University of Nottingham, Nottingham, UK; 2 Royal Derby Hospital, Uttoxeter Road, Derby, DE22 3DT, UK

**Keywords:** Extrema, Bloods pressure, Total peripheral resistance, Haemodialysis

## Abstract

**Background:**

Haemodialysis is a form of renal replacement therapy used to treat patients with end stage renal failure. It is becoming more appreciated that haemodialysis patients exhibit higher rates of multiple end organ damage compared to the general population. There is also a strong emerging evidence that haemodialysis itself causes circulatory stress. We aimed at examining haemodynamic patterns during haemodialysis using a new model and test that model against a normal control.

**Methods:**

We hypothesised that blood pressures generated by each heart beat constantly vary between local peaks and troughs (local extrema), the frequency and amplitude of which is regulated to maintain optimal organ perfusion. We also hypothesised that such model could reveal multiple haemodynamic aberrations during HD. Using a non-invasive cardiac output monitoring device (Finometer®) we compared various haemodynamic parameters using the above model between a haemodialysis patient during a dialysis session and an exercised normal control after comparison at rest.

**Results:**

Measurements yielded 29,751 data points for each haemodynamic parameter. Extrema points frequency of mean arterial blood pressure was higher in the HD subject compared to the normal control (0.761Hz IQR 0.5-0.818 vs 0.468Hz IQR 0.223-0.872, P < 0.0001). Similarly, extrema points frequency of systolic blood pressure was significantly higher in haemodialysis compared to normal. In contrary, the frequency of extrema points for TPR was higher in the normal control compared to HD (0.947 IQR 0.520-1.512 vs 0.845 IQR 0.730-1.569, P < 0.0001) with significantly higher amplitudes.

**Conclusion:**

Haemodialysis patients potentially exhibit an aberrant haemodynamic behaviour characterised by higher extrema frequencies of mean arterial blood pressure and lower extrema frequencies of total peripheral resistance. This, in theory, could lead to higher variation in organ perfusion and may be detrimental to vulnerable vascular beds.

## Introduction

Patients with end stage renal failure (ESRF) require renal replacement therapy (RRT). Haemodialysis (HD) is a form of RRT in which the blood is pumped from the patient into an extracorporeal circuit through an array of semipermeable membranes called the dialyser. The blood in the dialyser is separated from a fluid called the dialysate by a semipermeable membrane; and the plasma biochemistry changes towards that of the dialysate due to diffusion of the molecules according to their concentration gradients. Traditionally the treatment is given in three sessions a week each session lasting for three to four hours.

It is becoming more appreciated that HD patients exhibit higher rates of multiple end organ damage compared to the general population. HD patients for example have hugely elevated cardiovascular mortality which is estimated to be at least thirty times greater than aged matched population [1]. There is strong emerging evidence that HD in itself causes circulatory stress [2]. Many haemodynamic anomalies have been described during the HD process. Hypotensive episodes during haemodialysis complicate 20-30% of treatments [3,4]. These intradialytic hypotensive episodes were found to be predictors of mortality in HD patients [5,6]. Furthermore, regional myocardial hypoperfusion during haemodialysis causing regional wall motion abnormality (RWMA) has been demonstrated using both echocardiographic and perfusion scanning methods [7-9].

There are multiple factors that contribute to this abnormal cardiovascular function and complicate the HD treatment. On macrovascular level, vascular calcification is predominant in HD patients [10]. This results in increased arterial stiffness and reduces the ischaemic threshold in this population [11]. On microvascular level, endothelial dysfunction was evident in ex vivo experiments using wire myography [12] and laser Doppler imaging [13]. Furthermore, HD patients have higher rates of autonomic dysfunction expressed in studies as a reduction in baroreflex sensitivity (BRS) [14-16]. Intact BRS is essential for the regulation of blood pressure by activating compensatory mechanisms which keep the blood pressure within a narrow range [15].

All the above factors result in abnormal haemodynamic behaviour during HD that has not been fully characterised or correlated to measures of end organ damage. We aimed at examining the haemodynamic pattern during HD and at rest using a new model and test that model against a normal control.

## Materials and methods

### The haemodynamic model and its validation

Each heart beat generates a pressure pulsation expressed as systolic and diastolic pressures. These are largely dictated by the stroke volume and the compliance of the arterial tree. It is this compliance and distensability that lead to the damping of the pulse pressure in smaller arteries allowing tissue blood flow to be continuous throughout diastole [17]. From that point of view, perfusion pressure that is generated by each heart beat is expressed as the mean arterial blood pressure (MAP) [18]. MAP is not the arithmetic average of the systolic and diastolic pressures and is calculated in clinical practice as

(1) $$\text{MAP}\;=\;P_{\mathrm{Dia}}\;+\;\frac{P_{\mathrm{Sys}}-P_{\mathrm{Dia}}}{3}$$

The body uses several mechanisms such as local chemical and autonomic feedback to keep the perfusion pressure within the limits that are best for organ viability. We hypothesised that blood pressures generated by each heart beat constantly vary between local peaks and troughs (local extrema), the frequency and amplitude of which is regulated to maintain optimal organ perfusion. We also hypothesised that such model could reveal multiple haemodynamic aberrations in HD patients.

When measuring blood pressure by oscillometry using an inflatable cuff to occlude the brachial artery the measured systolic and diastolic pressures belong each to different heart beats. Hence, the use of such method would not provide sufficient resolution and the use of a continuous beat to beat cardiovascular monitoring device is needed to examine the above hypothesis.

The Finometer (Finapres Medical Systems, Arnhem, The Netherlands) is a tool for blood pressure and haemodynamic monitoring. It is particularly useful due to its non-invasive nature and ability to provide continuous readings over a period of several hours. The Finometer works by continuous pulse-wave analysis at the digital artery and utilises the finger-clamp method, in which changes in digital arterial diameter are detected by means of an infrared photoplethysmograph [19] and opposed by an ultra-fast pressure servo controller that changes pressure in an inflatable air bladder, both mounted in a finger cuff. This generates an arterial waveform that is measured on a beat-to-beat basis and is used to reconstruct a central aortic waveform by a validated transfer function [20]. This allows calculation of a full range of haemodynamic variables on a continuous basis; these include pulse rate (HR), mean arterial blood pressure (MAP), systolic blood pressure (SBP), diastolic blood pressure (DBP), stroke volume (SV), and total peripheral resistance (TPR).

Ethical approval was obtained from Nottingham research ethics committee (09/H0408/71).

Using the Finometer we recorded the above haemodynamic parameters in a HD patient and compared them with those of a healthy volunteer. The data was first recorded at rest for a period of 25 minutes for the HD patient and 30 minutes for the normal control (NC). Then the data was collected during a three hours HD session for the HD patient and to mimic the circulatory stress, the data for the healthy volunteer was collected during exercise using a cycling pedal for a similar period to the HD session. This yielded a total of 29,751 data points for each haemodynamic parameter measured between the two subjects for analysis.

**Figure 1:**
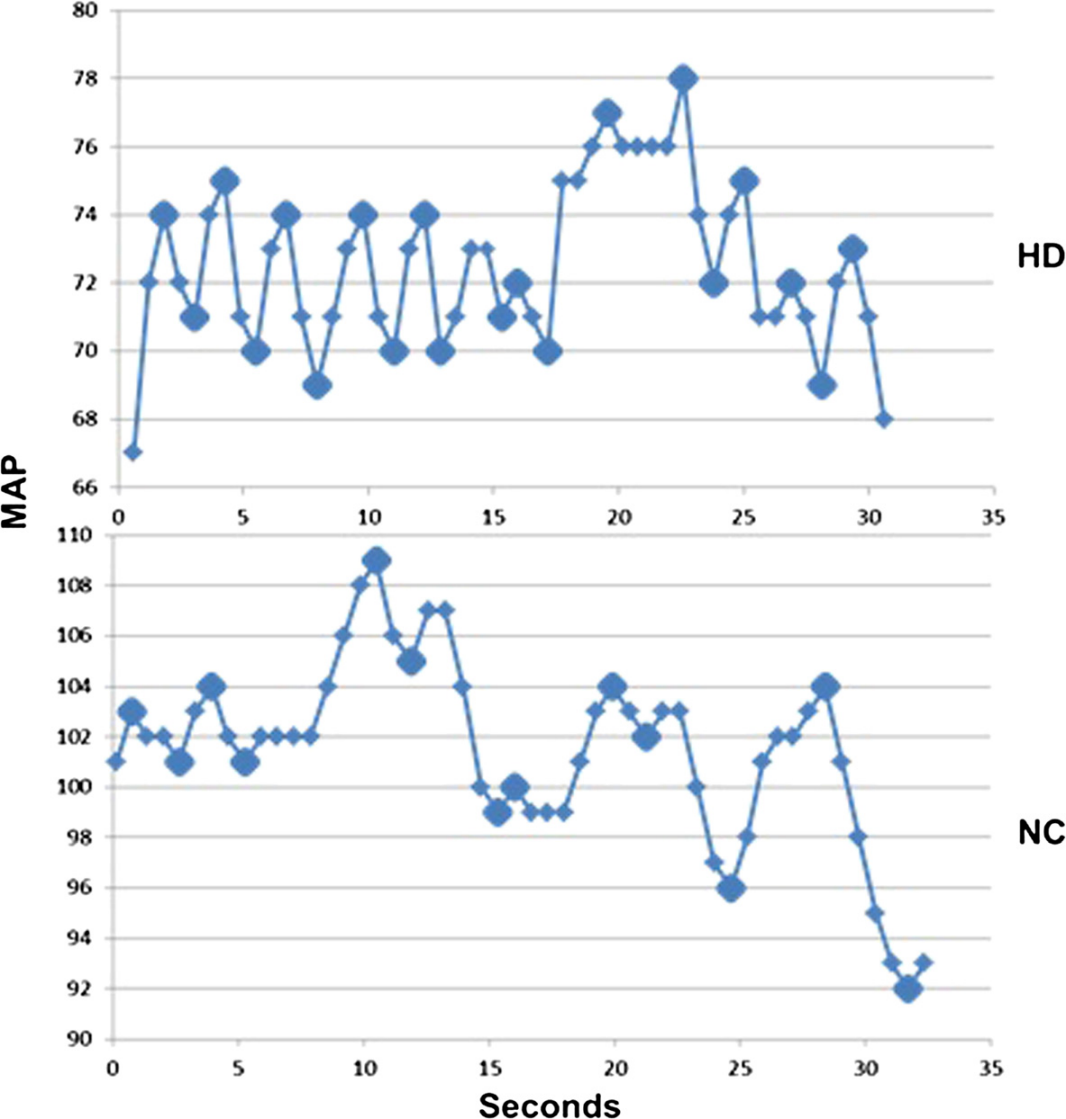
A 30 seconds sample demonstrating extrema points of Mean Arterial Blood Pressure (MAP) in a haemodialysis patient during haemodialysis (HD) and in a normal control (NC) during exercise.

The analysis of each haemodynamic parameter was done by identifying the local extrema points then calculating their frequencies and amplitudes using Matlab and Simulink (R2011a, MathWorks®, Natik, MA, USA). At first local extrema points were defined (local maxima and local minima). For the points (***P***) to qualify as an extrema it has to be either larger than both of its neighbouring points (maxima) or smaller than neighbouring points (minima) Figure 1.

(2) $$P_{\text{Maxima}}\;=\;P_{n-1}\;<\;P_{n}\;>\;P_{n+1}$$

(3) $$P_{\text{Minima}}\;=\;P_{n-1}\;>\;P_{n}\;<\;P_{n+1}$$

The extrema indices were also used to enable pairing with their time points (***t***). This was then used to calculate the time elapsed between each consecutive extrema.

(4) $$t\;=\;t_{\text{Extrema}\;+\;1}\;-\;t_{\mathrm{Extrema}}$$

The frequency (*F*) of the change between extrema points was then calculated.

(5) F=1/t

Finally, the amplitude (*a*) between two consecutive extrema points was also computed

(6) $$a\;=\;a_{\text{Extrema}+1}\;-\;a_{\mathrm{Extrema}}$$

### Computational implementation and statistical analysis

Computational implementation of the model was performed using Matlab and Simulink (R2011a, MathWorks®, Natik, MA, USA). Statistical analysis was performed using SPSS version 19 (IBM® SPSS Statistics, Inc.). An alpha error at 0.05 was judged to be significant. All continuous variables were tested for normality using their histograms and normality tests. Parametric data are presented by mean and standard deviation (SD), while non-parametric are presented as median and interquartile range (IQR). Between group differences were tested using the Independent *T* test or Mann U Whitney test depending on normality. Correlations were examined by a two-tailed Pearson test for parametric data and Spearman test for nonparametric data.

## Results

Characteristics of both HD and NC subjects are demonstrated in Table 1.

**Table 1:** Characteristics of normal control (NC) and haemodialysis (HD) subjects

	**HD**	**NC**
**Age**	68	37
**Gender**	Female	Male
**Diabetes Status**	Type II Diabetes Mellitus	None Diabetic
**Cardiovascular disease**	Stroke	None
**Blood Pressure (mmHg)**	121/75	118/78
**Haemodialysis Vintage (days)**	152	N/A
**Ultrafiltration Volume (mls)**	450	N/A
**Blood Flow (mls/min)**	400	N/A
**Type of Vascular Access**	Left Brachiocephalic Fistula	N/A

### Resting data

Extrema points frequency of MAP was higher in HD compared to that of NC in the resting state (0.781 Hz IQR 0.515-0.813 vs 0.337 Hz IQR 0.172-0.736, P < 0.0001). The same was noted for SBP and DBP as demonstrated in Table 2. The frequency of extrema points for TPR was lower in NC (0.787 Hz IQR 0.414-1.55 vs 0.829 Hz IQR 0.588-1.597, P < 0.0001) but with much higher amplitude (0.113MU IQR 0.039-0.239 vs 0.018MU IQR 0.01-0.031, P < 0.0001). Cardiac output extrema points frequency demonstrated the same pattern being higher in HD compared to NC at rest (0.804 Hz IQR 0.749-1.560 vs 0.786 Hz IQR 0.498-1.526).

**Table 2:** The frequency and amplitude of various haemodynamic parameters between haemodialysis (HD) patient and a normal control (NC) at rest

	**MAP**			**SBP**			**DBP**			**TPR**			**SV**		
	**HD**	**NC**	**P**	**HD**	**NC**	**P**	**HD**	**NC**	**P**	**HD**	**NC**	**P**	**HD**	**NC**	**P**
**Frequency**	0.781	0.337	<0.0001	0.788	0.419	<0.0001	0.763	0.508	0.002	0.829	0.787	<0.0001	0.793	0.727	<0.0001
	(0.0515-0.813	(0.172-0.736)		(0.536-0.821)	(0.234-0.795)		(0.401-0.818)	(0.239-1.518)		(0.588-1.597)	(0.414-1.55)		(0.539-1.493)	(0.391-1.470)	
**Amplitude**	5(3–6)	7(3–12)	<0.0001	8(6–10)	8(4–14)	0.549	3(2–4)	5(2–10.75)	<0.0001	0.018 (0.01-0.031)	0.113 (0.039-0.239)	<0.0001	10(6–13)	7(4–11)	<0.0001

Frequency (Hz), MAP (Mean Arterial Blood Pressure, mmHg), SBP (Systolic Blood Pressure, mmHg), DBP (Diastolic Blood Pressure, mmHg), TPR (Total Peripheral Resistance, MU), SV (Stroke Volume, ml).

### Stress data

Table 3 demonstrates the frequencies and amplitudes of all measured haemodynamic parameters during HD for the dialysis patient and during exercise for the NC. The frequency of change between extrema points of MAP was higher in the HD subject compared to the normal control (0.761 Hz IQR 0.5-0.818 vs 0.468 Hz IQR 0.223-0.872, P < 0.0001). These were independent of heart rate (r = 0.002, P = 0.887 for HD and r = 0.022, P = 0.290 for normal control). Similarly, extrema points frequency of systolic blood pressure was significantly higher in HD compared to NC (0.772 Hz IQR 0.541-0.840 vs 0.532 Hz IQR 0.331-0.851, P <0.0001) and so was cardiac output (0.80 Hz IQR 0.737-1.526 vs 0.786 HZ IQR 0.498-1.526, P < 0.0001). However, the frequency of the change between extrema points for TPR was higher in the normal control compared to HD (0.947 IQR 0.520-1.512 vs 0.845 IQR 0.730-1.569, P < 0.0001) with higher amplitudes (0.049MU IQR 0.022-0.106 vs 0.022MU IRQ 0.012-0.044, P < 0.0001) (Table 3).

**Table 3:** The frequency and amplitude of various haemodynamic parameters between haemodialysis (HD) patient and a normal control (NC) during circulatory stress

	**MAP**			**SBP**			**DBP**			**TPR**			**SV**		
	**HD**	**NC**	**P**	**HD**	**NC**	**P**	**HD**	**NC**	**P**	**HD**	**NC**	**P**	**HD**	**NC**	**P**
**Frequency**	0.761	0.468	<0.0001	0.772	0.532	<0.0001	0.766	0.777	0.052	0.845	0.947	<0.0001	0.78	0.743	<0.0001
	(0.5-0.818)			(0.223-0.872)	(0.541-0.840)	(0.331-0.851)		(0.403-1.477)	(0.311-1.479)		(0.739-1.569)	(0.52-1.512)		(0.565-1.470)	(0.478-1.41)	
**Amplitude**	4(3–6)	5 (2–9)	<0.0001	7 (3–11)	7 (5–10)	<0.0001	3 (2–50)	4 (2–8)	<0.0001	0.022 (0.012-0.044)	0.049 (0.022-0.106)	<0.0001	10 (6–15)	9 (6–13)	<0.0001	

Frequency (Hz), MAP (Mean Arterial Blood Pressure, mmHg), SBP (Systolic Blood Pressure, mmHg), DBP (Diastolic Blood Pressure, mmHg), TPR (Total Peripheral Resistance, MU), SV (Stroke Volume, ml).

On comparison with the resting data, circulatory stress seems to cause a large increase in the frequency of TPR extrema points in NC to a level that is higher than that of the HD subject (0.787 Hz IQR 0.414-1.55 vs 0.947Hz IQR 0.52-1.512, P < 0.0001). A similar but modest response in the frequency of TPR extrema points is noted in HD (0.829 Hz IRQ 0.588-1.597 vs 0.845 Hz IQR0.739-1.569, P = 0.023). Although there was an increase in the MAP extrema frequency when exercising in the NC (0.337 Hz IQR 0.172-0.736 vs 0.468 Hz IQR 0.223-0.872, P = 0.009) the frequency remains much lower than the HD patient which did not demonstrate a significant increase in the MAP extrema frequency during dialysis (0.781 Hz IQR 0.0515-0.813 vs 0.761 Hz IQR 0.5-0.818, P = 0.05).

## Discussion

In this proof of concept study we propose the use of frequencies and amplitudes of extrema points to analyse various haemodynamic parameters during HD. Testing the model during HD against an exercised normal control and at rest revealed multiple abnormalities in the haemodynamic behaviour of the HD subject. Firstly, we found that in the resting state the frequency of extrema points for MAP, SBP and DBP was higher in HD compared to NC. On the other hand, TPR extrema points frequency at rest was lower in NC but with higher amplitudes. As a response to circulatory stress the NC subject seems to have the ability to increase the frequency of TPR extrema points and maintain a much lower extrema frequency of MAP than the HD subject. This quicker change in TPR with higher amplitudes would in theory provide a better control and stability of blood pressure in the NC and the lack of this process in HD subject could be representative of autonomic dysfunction. HD patients as previously demonstrated have reduced baroreflex sensitivity which correlates with higher vascular calcification scores and increased arterial stiffness [14-16,21] and this could explain the differences between the two subjects at rest. However, the pattern of the haemodynamic response to circulatory stress seems to be less pronounced in HD compared to NC.

As MAP is considered the perfusion pressure for organs; in the HD subject the variation of MAP over one minute is higher than that of the normal volunteer. According to Ohm’s law the pressure difference between two ends of a vessel (P_1_, P_2_) is one of the determinants of blood flow

(7) $$Q\;=\;\frac{\left(\Delta P\right)}{R}$$

(8) $$\Delta P\;=\;P_{1}-P_{2}$$

Therefore, a higher variation in P_1_ and a lower variation in resistance (***R***) in theory would results in higher variation in blood flow to that organ, assuming that the venous pressure P_2_ is constant. Hence, in theory HD patients can experience a higher variation per minute in blood flow compared to normal exercised healthy volunteer. This haemodynamic behaviour could theoretically be detrimental to vulnerable vascular beds.

Previous studies that analysed haemodynamic parameters during HD used periodic averaging methodologies [22-24]. In the general population, other methods such as blood pressure variability using standard deviation or averaging the absolute values between adjacent readings have also been used [25,26]. Spectral analysis of blood pressure using Fast Fourier Transform formulae treats blood pressure readings as a signal and identifies the frequencies comprising it and their amplitudes [27]. Cowley et al. 1973 used distribution curves to demonstrate how blood pressure became very variable in baroreceptor-denervated dogs and was no longer maintained within a narrow range especially when stimulating the dogs [28].

We have shown using this model a marked difference in the haemodynamic behaviour at rest and in response to circulatory stress between the HD and the exercised NC subjects. The determinants of this haemodynamic behaviour in HD patients are still to be explored. Although these differences in haemodynamic performance could be attributed to differences in age and cardiovascular comorbidities between the two subjects; it is important to demonstrate the model’s ability to detect differences from two ends of the cardiovascular spectrum. The hypothesis that higher extrema frequencies of MAP and lower extrema frequencies of TPR during HD produces higher variability in organ perfusion; which could contribute to its damage is still to be tested. Studies that correlate between this haemodynamic model and measures of structural and functional organ damage are currently being undertaken.

## Competing interests

The authors declare that they have no competing interests.

## Authors’ contributions

MTE designed the model and its computational implementation, conceived the study, collected and analysed the data, and drafted the manuscript. AO conceived the study, collected and analysed the data, and drafted the manuscript. CWM conceived the study, critically reviewed the data analysis and drafted the manuscript. All authors read and approved the final manuscript.
